# Perception of the importance of chemistry research papers and comparison to citation rates

**DOI:** 10.1371/journal.pone.0194903

**Published:** 2018-03-28

**Authors:** Rachel Borchardt, Cullen Moran, Stuart Cantrill, Chemjobber, See Arr Oh, Matthew R. Hartings

**Affiliations:** 1 American University, NW, Washington, DC, United States of America; 2 Nature Chemistry, SpringerNature, London, United Kingdom; 3 Chemjobber, Shell, WV, United States of America; 4 Just Like Cooking, Krypton, KY, United States of America; Indiana University Bloomington, UNITED STATES

## Abstract

Chemistry researchers are frequently evaluated on the perceived significance of their work with the citation count as the most commonly-used metric for gauging this property. Recent studies have called for a broader evaluation of significance that includes more nuanced bibliometrics as well as altmetrics to more completely evaluate scientific research. To better understand the relationship between metrics and peer judgements of significance in chemistry, we have conducted a survey of chemists to investigate their perceptions of previously published research. Focusing on a specific issue of the *Journal of the American Chemical Society* published in 2003, respondents were asked to select which articles they thought best matched importance and significance given several contexts: highest number of citations, most significant (subjectively defined), most likely to share among chemists, and most likely to share with a broader audience. The answers to the survey can be summed up in several observations. The ability of respondents to predict the citation counts of established research is markedly lower than the ability of those counts to be predicted by the h-index of the corresponding author of each article. This observation is conserved even when only considering responses from chemists whose expertise falls within the subdiscipline that best describes the work performed in an article. Respondents view both cited papers and significant papers differently than papers that should be shared with chemists. We conclude from our results that peer judgements of importance and significance differ from metrics-based measurements, and that chemists should work with bibliometricians to develop metrics that better capture the nuance of opinions on the importance of a given piece of research.

## Introduction

Significance, importance and impact are all goals that the chemistry community strives to maximize—a breakthrough discovery, an important finding, a methodology that fundamentally changes the way chemistry research is performed. While individual researchers’ goals may vary greatly, many may aim to not only transform how chemistry research is performed, but to influence broader areas that transcend academic borders [1]. However, these abstract notions of significance, importance, and impact, particularly beyond the scholarly arena, are difficult to define, and even more difficult to quantify.

In recent years, many funding agencies have included requirements of efforts that move research outside of the laboratory. Among these is the U.S. National Science Foundation (NSF), which has recognized the importance of these goals with their “Broader Impacts” requirements in funding applications. The NSF gives guidance on these areas of impact, which include “Innovating for our future”, “Improving our society”, and “Engaging a wider audience” [2]. Efforts in these areas are meant to benefit society at large along with ensuring that chemistry (and other scientific disciplines) continues to receive popular support and maintains an ability to influence policy decisions.

A chemist’s work, then, includes the efforts expended in the laboratory on a research program as well as the efforts outside of experimentation for the benefit of society. Both types of work are necessary to further the field of chemistry. Therefore, it follows that chemists should be properly evaluated for the totality of their work. Some of the highest-stake evaluation points will occur early on during the career of an independent scientist: applying for tenure and seeking grant funding for new projects. Each of these evaluations is, essentially, a snapshot of a researcher’s research career, but can have very different aims. A tenure or promotion evaluation is generally reviewing a researcher for, among other criteria, impact and excellence in scholarship, while a grant funder is more interested in the potential of their research to have future impact. But what criteria are used to make these determinations about impact, excellence, and potential?

Relatively little insight is generally given into these evaluative processes, but it is well known within the chemistry community that citation-based metrics, most notably citation counts, impact factor, and h-index, play a large role in these evaluations [3,4]. Unfortunately, citation-based metrics may take some time before providing an estimation of the impact of a research program or an individual researcher. There is, therefore, a need to account for immediacy during these evaluations, particularly with grant funding.

Previous studies have found that grant decisions can be predicted, to some extent, by evaluation of h-index and similar metrics [5–7]. A recent study has called into question whether grant funding now serves as a “reward” for scientists who have accrued sufficient citation counts rather than as an award for promising research [8]. Another study found that h-index did not predict future success for junior level faculty [9].

Another area where perceptions of research significance plays a role is in the evaluation of individual manuscripts by journal editors and their referees. These decisions certainly reach beyond determining the potential of an article to generate citations. Editors evaluate significance and potential interest of an article along with how that manuscript fits into the scope of the journal. Referees assess the quality the experiments in an article, give their estimation on how well these experiments fit within the current research climate, and make general evaluations as to whether the importance of the research merits publication within the journal.

It is clear that traditional, citation-based metrics alone cannot be used to fully evaluate research or a researcher, but immediacy must be taken into consideration as part of these evaluations. It is also clear that the efforts of chemists to make their work relevant to society and achieve the goal of significance, as judged by their peers, have not been widely considered or measured.

Radicchi, Weissman, and Bollen recently looked at researchers’ perceptions of individual papers. They found that there was a low correlation between citation count and researchers’ perception of impact outside of instances where respondents were evaluating their own work [10]. The authors conclude that researchers are only capable of accurately measuring impact of studies in which they were involved. We, however, would postulate that their findings are an indication that citation counts may not be accurately measuring researchers’ abstract perception of impact within their field, but that this more abstract view of impact is instead replaced with citation counts when considering their own research. This takes into account that a researcher will likely be familiar with their own metrics, and have been evaluated on them in the past, while for other papers, they are more free to substitute their own definition of impact. Regardless, this disconnect between peer judgements of impact and citations for individual articles seems worthy of further exploration.

Accordingly, in this paper, we examine the broad concepts of significance and impact in order to better understand chemistry researchers’ perceptions of individual research articles. We evaluate how individual judgements, based upon different types of significance, relate to commonly-used citation-based metrics as well as other metrics. By doing so we show that researchers do have nuanced and measurable concepts of importance and significance in an attempt to understand how research and researchers might be evaluated when citation counts are not available or relevant. In effect, researchers are able to see different types of significance in an individual research article. We conclude by offering suggestions for how professional chemistry societies can work with bibliometricians to generate guidance for employing metrics that capture the entirety of a researcher’s efforts.

## Methodology

With the goal of testing the ability of chemists to evaluate research importance in mind, we chose an issue from the *Journal of the American Chemical Society* (volume: 125, issue: 31), published in 2003, 10 years prior to the survey date. This particular journal and issue was chosen to expose survey participants to articles in a wide variety of chemistry subdisciplines and topics from a flagship journal while controlling for journal-level impact, and to have allowed sufficient time for the articles to accumulate citations. We realize that there are a number of journals from which we could have chosen. Assisting in our decision to choose an issue from *JACS*, was that we all really liked the title, ‘*The JACS Challenge*’ for our survey. A survey instrument was developed and tested via personal correspondence with 25 chemists. Upon analysis of this initial data, noting the inability of professional chemists to correctly choose the 3 top cited articles, we decided to expand our efforts to include more respondents. This survey was then replicated via SurveyMonkey.

This study was exempted, after review, by American University's Institutional Review Board (Protocol #13167).

### Survey

The survey consisted of 8 questions, including basic demographic information such as research subdiscipline(s) and age. The following four questions were then applied to the same 63 articles from the JACS issue:

- Which three papers in the issue to you think are the most 'significant' (your own definition of 'significant' is what is important here)?
- Without looking up numbers, which three papers do you think will have been cited the most to-date?
- Which three papers would you most want to point out to other chemists?
- Which three papers would you want to shout about from the rooftops (i.e., tell anybody about, not just chemists)?

### Data

We recruited respondents using a number of blogposts and various solicitations on social media, including the blog ‘In the Pipeline’. In total, 408 people responded to the survey over a 2-week period. 45 sets of responses were removed for the following reasons: failing to give or denying consent on the IRB protocol form to use their answers for evaluation and submitting more than the requested number of responses (3) to a question. Removal of participants resulted in 363 participant responses included in our calculations.

Article citation counts for each article were recorded in November of 2013 and November of 2016 from Web of Science. H-index metrics of all corresponding authors were recorded in January of 2016 from Web of Science, and the highest h-index for each paper was used. Page Views were recorded from the journal’s website in November of 2016. Mendeley metrics were recorded from Mendeley in November of 2016.

The data set, available online via Figshare, was analyzed using Microsoft Excel [11].

### Methdology limitations

Our survey design and execution contain three main limitations. One, the participants in the survey were self-selected from those that are active within online chemistry communication channels, and are only a small subset of the entire field of chemistry. As such, they are not necessarily an accurate representation of the entire field, but their basic demographics are noted below. The second limitation is in the design of the survey itself–namely, that the four central questions were not order randomized. As a result, it is entirely possible that one question influenced the next. As shown in the results, most respondents chose different articles for the four questions, but the possible effects of this influence are unknown and unaccounted for. A third limitation is the open, rather than blind, manner in which the subdisciplines for each article were assigned through an evaluation by the authors of this paper.

We stress to the reader that this survey and study are designed to serve as preliminary analyses designed to illustrate and quantify abstract ideas in a new and interesting way. We invite bibliometricians and other researchers to more deeply delve into our data to examine additional trends not included in our study, including how self-citations may affect differences in total citations and perceived importance.

## Results and discussion

Table 1 lists the self-reported demographics of the respondents, who were able to select multiple subdisciplines to accurately and fully describe their training and current duties. The demographics of our respondents were weighted more heavily towards organic and medicinal chemistry than the general membership of the American Chemical Society [12]. We estimate that this is due to the readership of Derek Lowe’s blog ‘In The Pipeline’, which focuses on pharmaceutical chemistry and was likely the largest driver for publicizing this survey [13]. Nevertheless, we were able to attract respondents whose self-associated subdisciplines come from across the full spectrum of chemistry.

**Table 1:** Respondent demographics.

N = 363	Respondent Demographics								
Subdiscipline	Analytical	Biochemistry	Inorganic	Organic	Medicinal	Physical	Polymers	Other	Decline
Respondents	14	52	44	206	60	28	21	15	2
Papers Classified by Subdiscipline (63)	1	14	33	37	3	26	12	19	
Employment	Graduate Student	Postdoc	Faculty Member	Professional within Chemical Industry	Trained Chemist in a Non-Research Field	Decline			
Respondents	93	64	55	117	28	6			
Age	20–30	30–40	40–50	50–60	60–70	Above 70	Decline		
Respondents	140	141	54	23	2	0	3		

Each respondent was asked to choose 3 papers for each question. In looking at the papers that were selected, the highest correlation between the sets of answers was found for papers that were deemed significant and those that were thought to be the highest cited. Correlations among these categories are shown in Table 2. The correlation between papers deemed significant and those that should be shared with other chemists was higher than the correlation between those that were thought to be the most cited and those that should be shared with other chemists. This result seems to indicate that while chemists do equate significance with citations, the act of sharing papers with other chemists should also be tracked in more detail.

**Table 2:** Correlations between answers given for each question.

N = 363	Cited	Significant	Share: Chemists	Share: All
Cited	1	0.9	0.73	0.7
Significant		1	0.82	0.71
Share: Chemists			1	0.64
Share: All				1

The relationship between sharing with chemists and citations as well as between sharing with chemists and significance is certainly an interesting one. There may be certain papers which chemists find to be significant that might not be expected to show up in citation counts. Prior research focusing on the correlation between scientists storing and sharing papers (specifically through Mendeley reference libraries or over social media) and citation rate has shown that sharing can be predictive of future citations [14,15]. However, these earlier studies never directly looked at the evaluation of significance.

**Fig 1:**
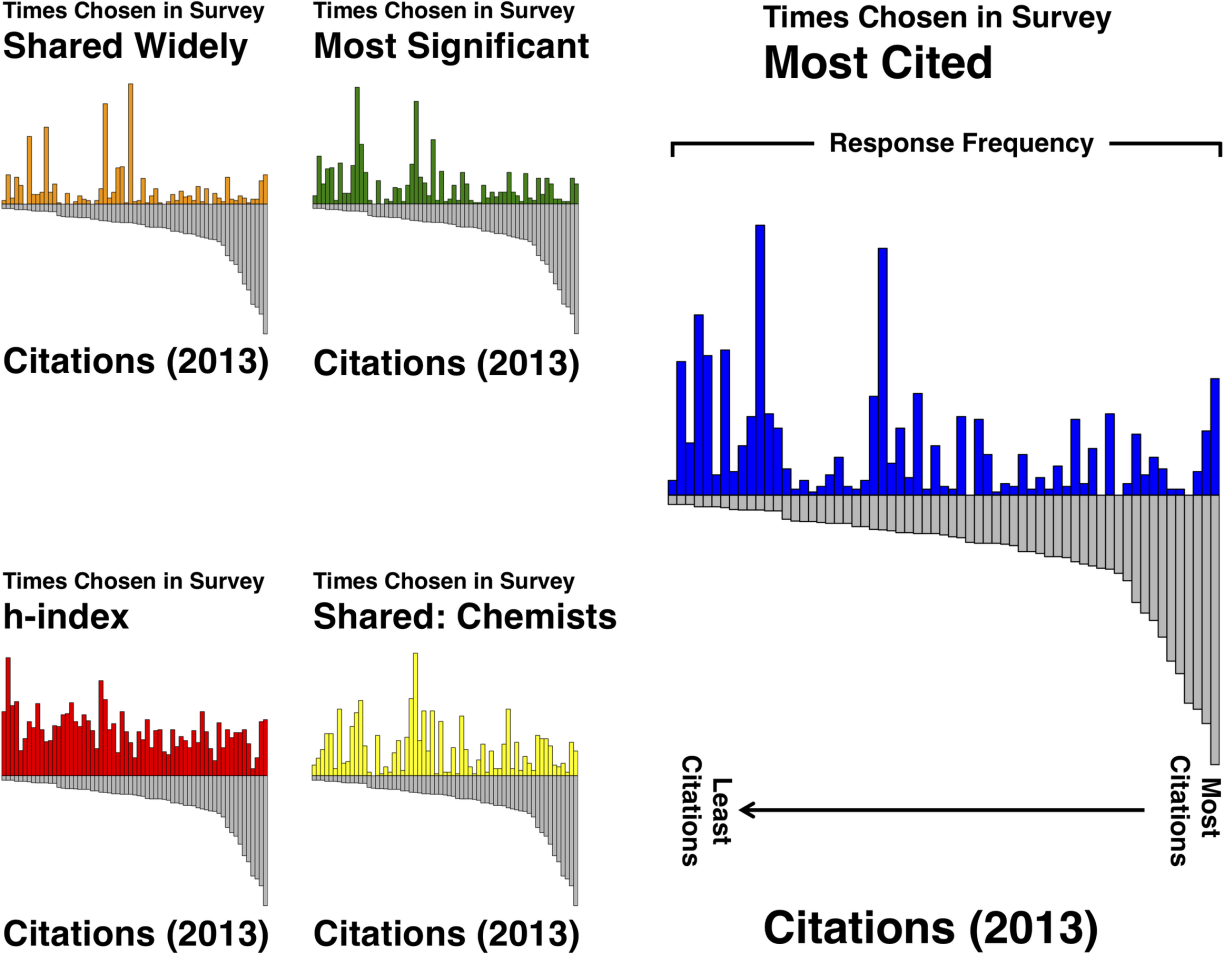
Respondent evaluations and citations (2013) by paper. The top panel shows the composite selections of our respondents for the question asking which papers they thought had the most citations (blue) and the actual number of citations in 2013 (gray). The other panels also include the number of citations (gray) along with: selections for most significant (green, middle left), selections for which should be shared with chemists (yellow, middle right), which should be shared widely (orange, bottom left), and h-index of the corresponding author (red, bottom right) for each of the manuscripts in the journal issue.

Because citations are the most common way in which the significance of a piece of research is broadly evaluated, we were interested in how well our respondents’ evaluations of citations, significance, and sharing correlated with the actual citations. Fig 1 shows several graphs that display total respondent selections with citations as of November of 2013 (when we closed the survey).

Focusing on the top panel (respondent evaluations of number of citations with actual citations), it is apparent that there are fluctuations in both the peer judgement of citation counts and actual citations. That is bound to be expected as there are definitely variations in citation rate, even within journals. Stuart Cantrill, one of the authors on this paper and chief editor of *Nature Chemistry*, has commented on this phenomenon in the context of that journal’s JIF, noting the contribution of a small handful of highly cited papers to their overall impact factor, with the majority of articles receiving fewer citations than the impact factor would predict [16]. This phenomenon also led to a published proposal by several leading scientific publishers for the adoption of citation distributions as a replacement for JIF [17].

**Fig 2:**
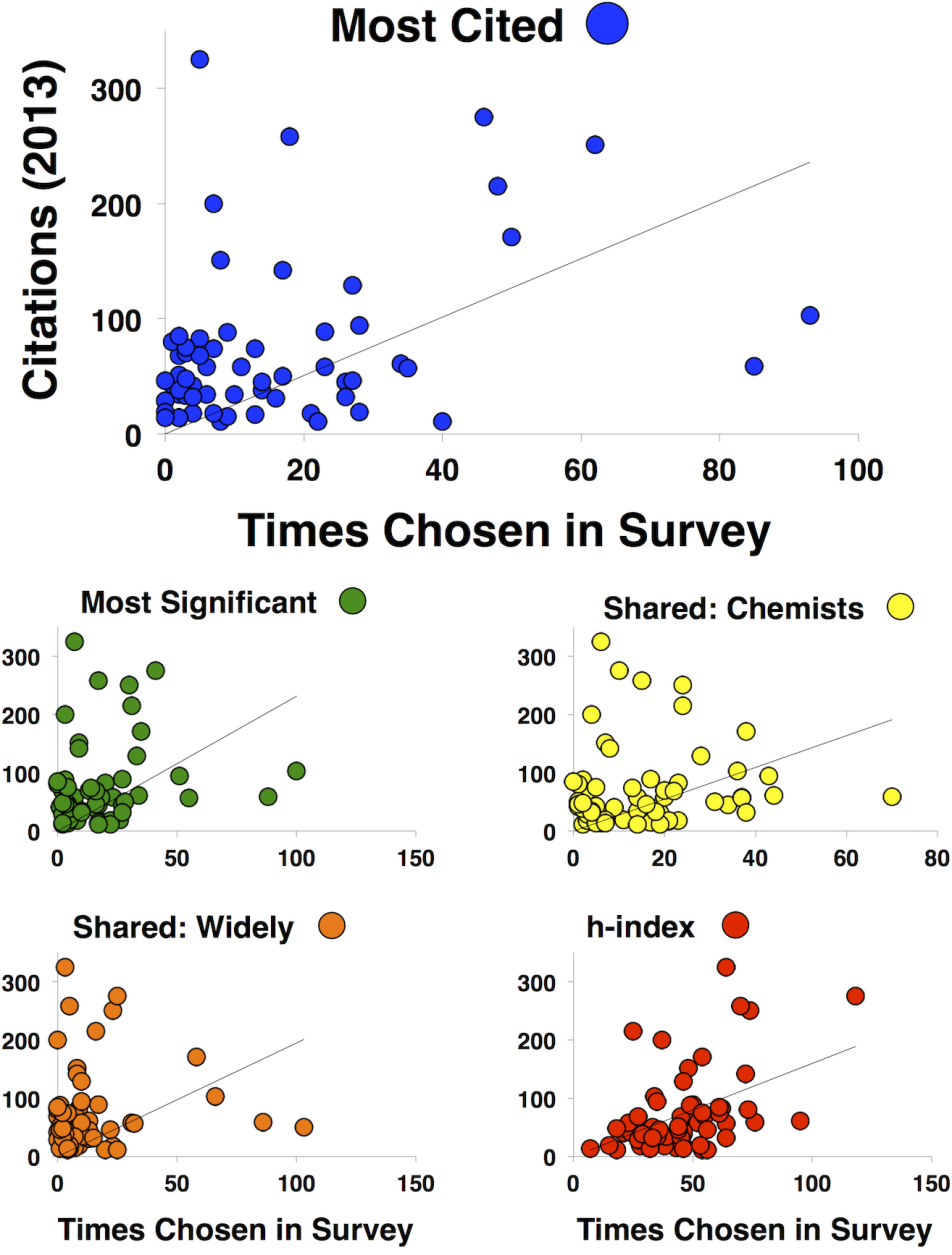
Correlations between respondent choices and citations (2013). The top panel is a graph plotting each papers number of actual citations (2013) versus the number of times it was chosen by our respondents for the question: ‘which three papers do you think have been cited most to-date’ (blue). The other panels also plot actual citations versus survey responses: most significant (green, middle left), shared with chemists (yellow, middle right), and shared widely (orange, bottom left). The number of actual citations versus the h-index of the corresponding author is shown in the bottom right panel (red).

We see the same kind of fluctuations in the evaluations of our respondents. What is interesting to us are the differences in what the respondents expect to be well-cited papers versus which papers are actually well cited. Fig 2 is a more direct way to view the correlation between respondent choices and actual citation counts.

To put these graphs in perspective, we calculated the correlation between the aggregate responses for individual survey questions and the actual citations from both 2013 (the year the survey was taken) and 2016. As shown in Figs 1 and 2, there were a number of papers that were selected disproportionately more than these same papers were actually cited. We were curious if that might be because the respondents were viewing the ‘leading edge’ of chemistry. That is, if these articles were very cutting edge, it might take their citation counts a longer time to build up, like the noted Sleeping Beauty papers [18]. The data doesn’t support this point of view as the correlations, generally, decrease from the 2013 citations to the 2016 citations.

Delving into the individual survey questions, we found generally low correlations between each of the four categories and citations, as well as the correlation with the corresponding author’s h-index, as shown in Table 3. There are several observations that can be made from these comparisons. Primary among these is that corresponding author h-index has a stronger correlation than any of our survey responses to actual citation counts. In some ways, this is unsurprising since both h-index and impact factor play a role in citation count, and h-index is sometimes the strongest predictor of citation count [19,20]. We would caution that h-index and citation count don’t fully capture the notion of significance, as measured by our survey, indicating that, despite their common use in evaluation, true significance cannot be captured solely by these commonly-used bibliometrics. However, our findings also concur with one study in chemical engineering that shows that author and research group name recognition correlate strongly with citation count, which gives more evidence to the idea that peer judgments are influenced by name recognition [21].

**Table 3:** Correlation between respondent choices and citation counts from 2013 and 2016.

Correlation with citations (2013)				
Cited	Significant	Share: Chemists	Share: All	h-index
0.33	0.2	0.06	0.10	0.47
Correlation with citations (2016)				
Cited	Significant	Share: Chemists	Share: All	h-index
0.3	0.19	0.06	0.08	0.47

On the subject of true significance, it is worth noting that the survey responses indicate far from perfect agreement. While general trends are certainly demonstrated, the chemists did not all select the same articles for these different proxy measures of abstract importance and significance. However, the fact that there are trends at all show that there is a level of general agreement. Regardless, we posit that human judgement, even limited in scope as presented in these results, is a more accurate reflection of the abstract notion of significance upon which we can then compare metrics such as citation count. We furthermore recognize that the true value of a single article to the academic community as well as society as a whole can probably never be fully represented by any human or metric measures.

The second noteworthy observation from these numbers is that our respondents’ judgements of citation numbers were better correlated to actual citations than their estimations of other qualities (significance, shared with chemists, and shared broadly). In some way, this is reassuring; in comparison to the other metrics in the survey, chemists are best at judging citations. This further supports the hypothesis that our respondents, collectively, seem to view citations as being different than significance.

Curious, to us, is the correlation between the articles that would be shared with other chemists and the actual citation count. For the comparison to the citation counts in 2013, the 'shared with chemists' has the lowest correlation of all of the evaluations in our survey. As a citation is literally a way that scientists recommend articles to one another, it is surprising to us that its correlation to actual citation counts isn’t slightly higher. One reason for this might be due to the fact that our respondents might equate ‘shared with chemists’ with a paper being ‘interesting’ rather than ‘useful’. For the sake of this argument, it is instructive to think of two kinds of novelty produced by chemical research: results that lead to a new way of thinking about chemistry and results that lead to a new way of doing chemistry. The latter include reports that are often defined as being ‘methods’ papers. Our results, when viewed from this perspective may be hinting that there might be recognizable differences between a citable paper and a thought-provoking paper. This conclusion is supported by Li and Thelwall, who observed differences in the prediction strength of F1000 peer judgements for different types of articles, as well as other researchers who note that Mendeley statistics could measure “hidden impact” of some types of research articles [22,23]. While the present study does not fully answer this question, it would be useful to study how chemists view and engage with research that explicitly fall squarely into different categories. These results also hint at the importance altmetrics could play, more generally, in capturing this type of significance that citations alone cannot. Unfortunately, due to the age of the journal issue in question, we were unable to track these articles using altmetrics evaluation tools to confirm whether these papers were, indeed, shared widely through social-media channels.

From our full set of results, we wanted to find out how much the selections made by respondents were dictated by their identification with a specific subdiscipline of chemistry. Before we could do this, we had to determine which subdisciplines each of the papers could accurately be assigned to. Individually, the four authors with graduate training in chemistry (SC, CJ, SAO, and MRH) evaluated each article for their judgments on each article’s subdisciplines. The final decision on subdiscipline was made through a collaborative discussion between these authors. All chemical research relies on influence and techniques that come from multiple subdisciplines. However, we made our decisions based on which subdisciplines most informed a particular study as well as which subdiscipline might be most affected by that study. For an article, any subdiscipline that was suggested by our evaluations was accepted. By our analysis, only 4 articles were described as falling within a single subdiscipline, 37 articles within 2, 21 articles within 3, and a single article designated to 4 subdisciplines.

Table 4 shows correlations that analyze the effects of subdiscipline on respondent choices. First, we looked at how often respondents made selections within their own discipline. Papers that were thought to be highest cited and most shared with chemists were selected from the respondent’s subdiscipline 70% of the time. Papers that were thought to be significant were selected from a respondent’s subdiscipline slightly less often, at 63%.

**Table 4:** Analysis of selections within and outside of respondents’ subdisciplines.

	Were papers chosen from within respondent’s discipline? **Yes.** Percentage of articles chosen from respondent’s discipline			
	Cited	Significant	Share: Chemists	Share: All
	69	63	71	51
	Were paper choices made within discipline more accurate than those that came from the respondents with a different discipline? **Not Really.** Correlation between choice and actual citations			
	Cited	Significant	Share: Chemists	Share: All
In Discipline	0.28	0.18	0.02	0.17
Out of Discipline	0.27	0.12	0.11	-0.01

**Fig 3:**
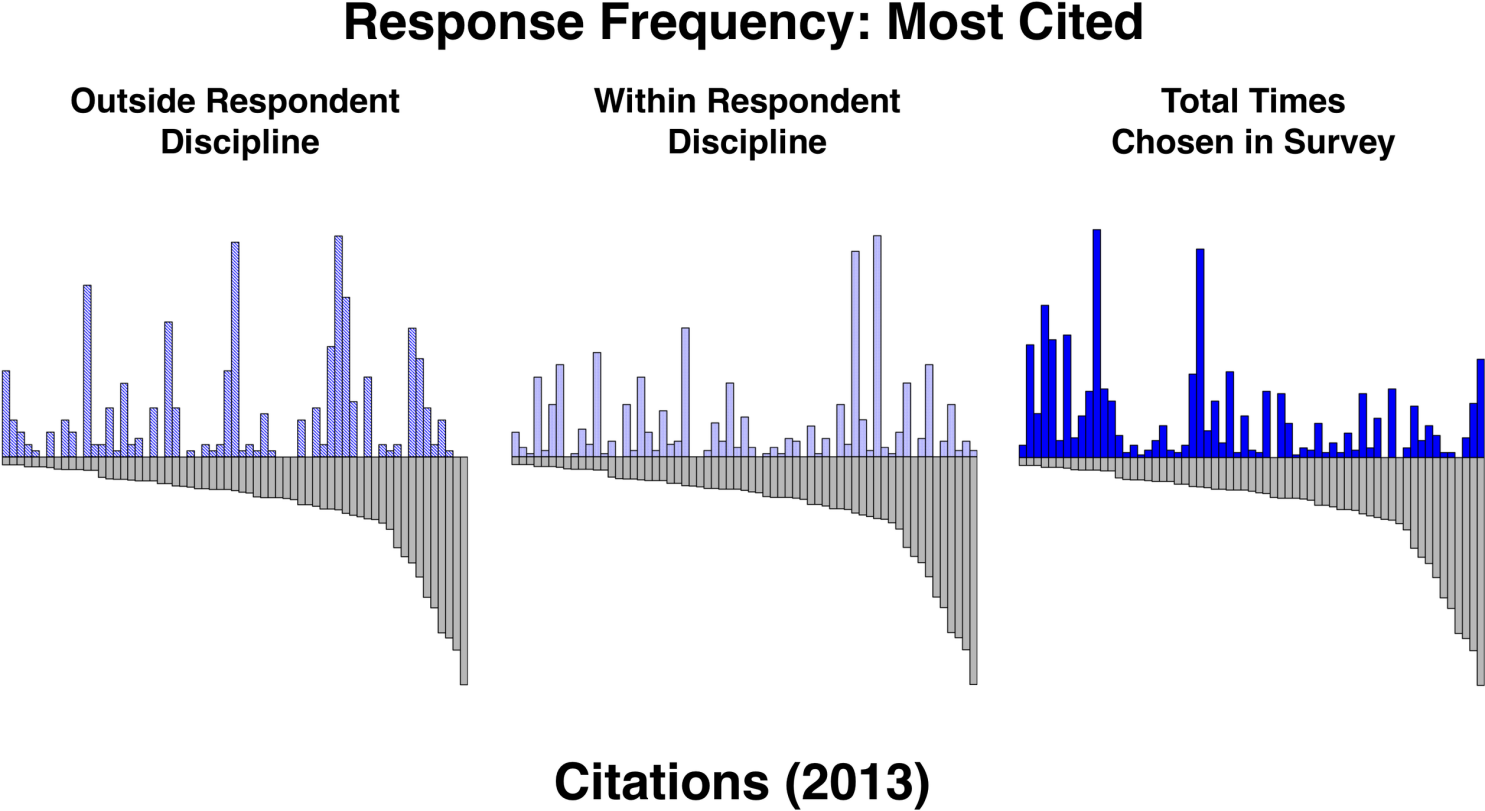
Respondent selections of most cited by subdiscipline. Each graph shows respondent selection for most cited (left axis, blue) and actual citations as of 2013 (right axis) for each paper. The top panel shows selections from all respondent subdisciplines. The middle panel shows selections where the respondent subdiscipline matches the article subdiscipline. The bottom panel shows selections where the respondent subdiscipline does not match the article subdiscipline.

We also looked to see if selections within subdiscipline correlated better with actual citations than selections outside of subdiscipline. Selections from within subdiscipline and outside of subdiscipline had the same correlation to actual citations in 2013, and both sets had a lower correlation than the aggregate selections. Looking at the paper-by-paper data (Fig 3), there are examples of individual subdisciplines’ ability, or lack thereof, to correlate to actual citations.

In some cases, the respondents’ choices of times cited had a strong correlation to actual citations. Paper 45, ‘A Heterocyclic Peptide Nanotube’ was cited 171 times as of 2013. This paper was selected as being highly cited 50 times, 42 of which coming by respondents that self-identified as having the subdisciplines that characterized that paper. Paper 10, ‘Single-Step *in Situ* Synthesis of Polymer-Grafted Single-Wall Nanotube Composites’ was cited 251 times as of 2013. Our respondents chose this paper as being highly cited 62 times, 34 times from within the subdiscipline and 28 times from outside of the subdiscipline. Paper 5, ‘An Ionic Liquid-Supported Ruthenium Carbene Complex: A Robust and Recyclable Catalyst for Ring-Closing Olefin Metathesis in Ionic Liquids’ was cited 215 times as of 2013. Our respondents chose this paper 48 times, 13 times from within the subdiscipline and 35 times from outside of the subdiscipline.

In some cases, our respondents entirely underestimated the citation count of an article. Paper 43, ‘Factors that Determine the Protein Resistance of Oligoether Self-Assembled Monolayers—Internal Hydrophilicity, Terminal Hydrophilicty, and Lateral Packing Density’ was cited 325 times as of 2013. Our respondents chose this paper a total of 5 times, all from within the paper’s subdiscipline.

In some cases, our respondents over-estimated the citation count of an article. Paper 30, ‘Formation of Nanostructured Polymer Filaments in Nanochannels’ was only cited 11 times as of 2013. Our respondents chose this paper 40 times, 36 times from outside of the papers subdiscipline. Paper 18, ‘TNA Synthesis by DNA Polymerases’ was cited 59 times as of 2013. Our respondents chose this paper 85 times, 72 times from within the paper’s subdiscipline.

What this data indicates to us is that human-based prediction and judgement of citation rate is difficult. As seen in other fields looking at the predictive power of F1000, active input from a broadly represented group of chemists is needed to best estimate the citation-impact of an article. But even then, it should be expected that the group will overestimate some papers while underestimating others, as echoed in previous studies [24,25]. Yogi Berra, a baseball player who is noted for his quotability as much as his skill, once said, “It’s tough to make predictions, especially about the future.” Our study shows that it can be just as tough to make predictions about the past.

Carrying forward the ability of diverse perspectives to optimize judgements and predictions, we wanted to see if the aggregate responses to all of our survey questions could better judge citation counts. Table 5 shows the correlations of combinations of responses to 2013 citation counts. What we find from this analysis is that, based upon the questions we asked of our respondents, questions about citation counts were best at actually choosing more highly-cited articles.

**Table 5:** Correlation of aggregate survey responses to 2013 citation counts.

Correlation to Citations in 2013			
All Selections: Cited + Significant + Share: Chemists + Share: All	Cited + Significant + Shared: Chemists	Cited + Significant	Cited
0.2	0.22	0.27	0.33

We next looked at the correlation between metrics not commonly used in research evaluation and citation counts in relation to our peer judgements. For this study, we have chosen to look at two metrics: inclusion in Mendeley libraries and page views. One reason we chose to look at these metrics is their immediacy–that is, that these metrics can both be gathered and analyzed relatively quickly following an article or even pre-print publication, and thus serve as interesting potential metrics for the evaluation of junior level researchers. Additionally, as mentioned before, they may hold promise in being able to give more nuanced views of impact. Table 6 shows the correlations between these metrics with citation counts and the responses from our survey. As can be seen with the correlations to the 2013 and 2016 citations, both the Mendeley and page-views metric are better at matching relative citation counts than any of our survey responses, reaffirming previous studies that have found correlations between these metrics. These two metrics also show higher correlations to citation rates than does the h-index of the corresponding author. We would like to caution that correlations to these metrics (as we present them) are not predictive and are, likely, formed somewhat in response to the ways these papers are cited. However, what is apparent is that even these metrics fail to replicate what practicing chemists view as significant and important, indicating that, at least for Mendeley, it alone cannot adequately capture these more elusive properties.

**Table 6:** Correlations between Mendeley inclusions or page views with citations and survey results.

Correlation to Mendeley inclusions					
2013 Citations	2016 Citations	Most Cited	Most Significant	Share: Chemists	Share: All
0.75	0.76	0.29	0.22	0.17	0.19
Correlation to page views					
2013 Citations	2016 Citations	Most Cited	Most Significant	Share: Chemists	Share: All
0.83	0.85	0.31	0.24	0.20	0.24

## Conclusions and outlook

While the analysis of article citations will always be a necessary activity, it is apparent that citation count does not necessarily correlate with perceived importance. Or, put better: that the importance of a research article is only partly captured by its citation rates. This is certainly not a new finding. But we do hope that our study reinforces previous studies and pushes for use of a better metric or set of metrics that captures paper importance that reflects objective analyses (usage of the paper through citation rates and implementation of its findings outside of the research lab) as well as the more subjective point-of-view of active researchers. Returning to the distinction between papers that change the practice of chemistry versus those that change a chemist’s point of view, how do researchers engage with these distinct types of research articles? Is there a way to capture nuances of how researchers perceive a research article? Could altmetrics play a role in telling this side of the story? Our results indicate that in order to more closely align current measurements with peer judgement, perhaps the future of chemistry impact evaluation should rely more heavily on perception, peer evaluation, and other qualitative peer judgements than on bibliometrics, in addition to those bibliometrics and altmetrics currently available.

Another aspect that continues to motivate us involves the immediacy of research. A full accounting of the importance of a paper, however importance is defined, cannot be accurately evaluated until long after the research has been published. However, scientists are judged on the perceived potential of their work before it occurs (while evaluating proposals for funding) and directly after a set of experiments is completed (refereeing manuscripts for journals). The inability of our panel of experts in chemical research to outperform h-index in judging journal citations suggests the views of just a few critical reviewers: journal editors, program officers, article and grant referees, and the scientists performing the work are inadequate for accurately capturing the importance of a research project and result. Perhaps there are other leading indicators, such as engagement with an article on a preprint server, that can better predict and evaluate the importance of a research project prior to publication, provided that a diverse and robust group of peers can be relied on to engage in such activities in an online sphere.

With these findings, we conclude that the time has come for the chemistry community to actively seek better measures for the evaluation of researchers in the context of the broader impacts we describe earlier in the paper. Our suggestion is not a new one, but adds to calls made by others in chemistry and beyond [26–30]. We note that several other disciplines have already investigated and articulated the need for broader impact measures. The most well-defined example of a model for the evaluation of research based on broader impacts is the Becker Model [31]. The Becker Model outlines the impact of research output and activities in five distinct areas of impact: Advancement of Knowledge (which generally corresponds to traditional scholarly impact), Clinical Implementation, Legislation and Policy, Economic Benefit, and Community Benefit. For each area of impact, it includes a list of different research outputs and activities, as well as measures and other indicators of impact. Other examples include practitioners of marketing and policy research, who have suggested ways to track and foster ‘societal impact’, and the development of a task force in academic librarianship, who are charged with creation of a framework for the evaluation of scholarship in a field that is generally considered to be practitioner-driven [32,33].

We specifically call on all international chemical societies to work together to create an evaluative framework for chemistry researchers that go beyond commonly-used bibliometrics to create a framework for the equitable evaluation of research in the context of the broad impacts it provides to scholarship and in the greater society.
